# Gene Expression Profiles Associated with Radio-Responsiveness in Locally Advanced Rectal Cancer

**DOI:** 10.3390/biology10060500

**Published:** 2021-06-03

**Authors:** Jeeyong Lee, Junhye Kwon, DaYeon Kim, Misun Park, KwangSeok Kim, InHwa Bae, Hyunkyung Kim, JoonSeog Kong, Younjoo Kim, UiSup Shin, EunJu Kim

**Affiliations:** 1 Division of Radiation Biomedical Research, Korea Institute of Radiological and Medical Sciences, Seoul 01812, Korea; jeeyongl@gmail.com (J.L.); kimd1106@daum.net (D.K.); kskim@kirams.re.kr (K.K.); ihbae@kirams.re.kr (I.B.); 2 Department of Radiological & Clinical Research, Korea Cancer Center Hospital, Korea Institute of Radiological and Medical Sciences, Seoul 01812, Korea; jhkwon@kirams.re.kr (J.K.); usre@kirams.re.kr (M.P.); hk0811@kirams.re.kr (H.K.); younjoo282@gmail.com (Y.K.); 3 Department of Radiological and Medico-Oncological Sciences, University of Science and Technology, Daejeon 34113, Korea; 4 Department of Pathology, Korea Cancer Center Hospital, Korea Institute of Radiological and Medical Sciences, Seoul 01812, Korea; balltta9@kirams.re.kr; 5 Department of Internal Medicine, Korea Cancer Center Hospital, Korea Institute of Radiological and Medical Sciences, Seoul 01812, Korea; 6 Department of Surgery, Korea Cancer Center Hospital, Korea Institute of Radiological and Medical Sciences, Seoul 01812, Korea

**Keywords:** rectal cancer, radiation therapy, radio-responsiveness, bio-marker, DNA methylation

## Abstract

**Simple Summary:**

Standard treatment of locally advanced rectal cancer (LARC) consists of chemotherapy, radiotherapy, and surgery. Identification of radio-resistant (RR) and radio-sensitive (RS) LARC has been a major hurdle for patient-specific treatment. The development of biomarkers that can discriminate radio-responsiveness before surgery could improve standard treatment and minimize unwanted side effects.

**Abstract:**

LARC patients were sorted according to their radio-responsiveness and patient-derived organoids were established from the respective cancer tissues. Expression profiles for each group were obtained using RNA-seq. Biological and bioinformatic analysis approaches were used in deciphering genes and pathways that participate in the radio-resistance of LARC. Thirty candidate genes encoding proteins involved in radio-responsiveness–related pathways, including the immune system, DNA repair and cell-cycle control, were identified. Interestingly, one of the candidate genes, cathepsin E (*CTSE*), exhibited differential methylation at the promoter region that was inversely correlated with the radio-resistance of patient-derived organoids, suggesting that methylation status could contribute to radio-responsiveness. On the basis of these results, we plan to pursue development of a gene chip for diagnosing the radio-responsiveness of LARC patients, with the hope that our efforts will ultimately improve the prognosis of LARC patients.

## 1. Introduction

Colorectal cancer (CRC) is the third-most common type of cancer globally, constituting ~10% of all cancer cases [1]. Over the past 20 years, the 5-year survival rate for CRC patients has improved owing to advances in surgery, radiotherapy, chemotherapy, and overall healthcare systems, yet CRC still ranks high in mortality, reflecting its tendency to progress without symptoms until quite advanced. CRC can be categorized into colon and rectal cancers. While rectal and colon cancers are similar in many aspects, their treatments are quite different. The tight quarters in the rectum and proximity to other organs and structures can make surgery to remove rectal cancer a very complex undertaking. Conventional treatments for locally advanced rectal cancer (LARC) include combined surgery, chemotherapy, and radiotherapy [2]. A report from a German trial in 2004 recommended a standard treatment composed of neoadjuvant chemo-radiation therapy (NCRT) followed by radical surgery with total mesorectal excision (TME) [3,4]. The inclusion of NCRT has a beneficial effect in reducing the rate of local recurrence and enhancing the rate of cancer survival [5,6,7].

Currently, one-sixth of patients are reported to show a pathological complete response (pCR) before radical surgery [8], but despite clinical observation of pCR, radical resection is still performed. If NCRT could eradicate all cancer cells in pCR cases, radical surgery might not be needed. Omitting the surgical resection process could avoid unnecessary surgical morbidity and impaired life quality. In this regard, Gamma et al. proposed that if strict endoscopic criteria are applied in determining pCR, delayed or even no surgery could be more beneficial for the patients [9]. In fact, a recent report indicated that this ‘wait and see’ approach does not compromise long-term oncological results [10], suggesting that amending the standard treatment to include this ‘wait and see’ feature would allow patients to avoid unnecessary radical surgery and its associated surgical morbidity and impaired life quality. On the other hand, since ~25% of patients would not benefit from NCRT [8], more intense treatments could be used on these NCRT non-responders. Cytotoxic agents (5-fluorouracil, oxaliplatin, and irinotecan), epidermal growth factor receptor (EGFR) inhibitors (cetuximab and panitumumab), and vascular endothelial growth factor (VEGF) inhibitors (bevacizumab and aflibercept) are usually added to enhance the radiation response. However, simple inclusion of these treatments for all LARC would represent overtreatment for certain patients. Therefore, there is a need for improved standard treatments for LARC that could benefit these patients. 

Precision medicine has received considerable recent attention as an innovative approach for cancer treatment [11]. The heterogeneity of cancers often demands case-by-case treatment decisions. Selecting the most effective treatment for individual patients absolutely requires a precise analysis of the disease condition. Researchers have sought to categorize high- and low-risk patient groups based on clinical imaging technologies and pathological prediction [12]. However, these efforts have not proved capable of fully discriminating cases with poor prognosis. Other researchers have devoted their efforts to identifying molecular biomarkers and have found a number of such potential predictors, including genomic instability, K-Ras, TP53 and DCC, among others [13]. However, factors identified to date have provided limited clinically applicable information. Among CRC types, LARC in particular could be an ideal candidate for clinical application of precision medicine. Standard treatments for LARC could be modified according to the expected radio-responsiveness of the patient’s tumor. For example, if pCR was indicated for a given patient, invasive radical surgery could be avoided. On the other hand, for tumors predicted to have poor radio-responsiveness, more intensive chemotherapy could be applied before and/or after the surgical operation. Therefore, the development of biomarkers capable of successfully assessing patients’ radio-responsiveness status is urgently needed to establish patient-specific treatment [14]. 

To address this need, we sorted LARC patients according to their radio-responsiveness and generated organoids from the corresponding cancer tissues. Organoids have proven to be a good model system for investigating various cancers, especially gastrointestinal tissues [15]. Next, we categorized the organoids into two groups based on their responsiveness to radiation. We then obtained expression profiles for each group, after which we used biological and bioinformatic methods to analyze the candidate genes and pathways that contribute to radio-responsiveness in LARC. We also focused on differential DNA methylation at the promoter region, testing its association with radio-responsiveness. Our hope is that the biomarker candidates identified here will aid in the application and effectiveness of radiation therapy, enabling LARC patients who are not candidates for surgery or whole-body chemotherapy to receive this relatively safe therapy, and ultimately experience an improved quality of life after treatment.

## 2. Materials and Methods

### 2.1. Patient Enrollment and Treatment

Six pathologically proven LARC patients, treated from April 2018 to May 2019, whose NCRT treatment outcomes were categorized as complete response (n = 3) or poor response (n = 3) based on total mesorectal excision specimens were selected for inclusion. Staging workups consisted of pelvic magnetic resonance imaging (MRI); computerized tomography (CT) of the chest, abdomen, and pelvis; and 18-fluoro-2-deoxy-glucose positron emission tomography (PET)/CT scans. NCRT was performed using a long-course protocol consisting of a 50.4 Gy dose delivered in 28 fractions on weekdays. Chemotherapy was performed as single-agent 5-fluorouracil (425 mg/m^2^), infused for 5 days every 4 weeks before surgery. Radical surgeries were performed ~6–8 weeks after completion of radiotherapy. All surgeries were performed according to the principle of total mesorectal excision. 

The pathologic response after NCRT was evaluated using the tumor regression grade (TRG) system suggested by the Gastrointestinal Pathology Study Group of the Korean Society of Pathologists [16]. Tumors were defined under the TRG system as follows: Grade 0, complete response—no residual tumor cells identified; Grade 1, near-complete response –tumor bed contains abundant fibrosis with only a few or scattered tumor cells; Grade 2, partial response—residual tumor glands are easily identified in the tumor bed; Grade 3, poor or no response. 

### 2.2. Tissue Acquisition

Pre-NCRT rectal cancer and normal biopsy samples were obtained from enrolled patients before NCRTs. Four or five biopsy samples were collected from endoscopic cancers or normal mucosa of the rectum. Collected samples were verified histologically as adenocarcinoma or normal crypt based on an analysis of hematoxylin and eosin (H&E) stained tissue by a pathologist. Biopsy samples were pooled and immediately placed in cold phosphate-buffered saline (PBS) containing gentamicin. 

### 2.3. Organoid Culture 

Patient-derived organoids were isolated as described previously [17]. In brief, cancer tissues were incubated in collagenase type II (Sigma-Aldrich, St. Louis, MO, USA), dispase type II (Roche Applied Science, Mannheim, Germany) and Y-27632 (BioVision, Mountain View, CA, USA) for 30 min at 37 °C. The cells were then embedded in Matrigel on ice (growth factor reduced, phenol red free; Corning, Inc., Corning, NY, USA) and seeded in 24-well plates, followed by addition of culture medium. The patient-derived organoid culture medium contained 1xB27 (Gibco, Grand Island, NY, USA), 1.25 mM N-acetyl cysteine (United States Pharmacopeia, Rockville, MD, USA), 50 ng/mL human epidermal growth factor (BioVision), 50 ng/mL human Noggin (Peprotech, Rocky Hill, NJ, USA), 10 nM gastrin (Sigma-Aldrich), 500 nM A83-01 (BioVision) and 100 mg/mL primocin (InvivoGen, San Diego, CA, USA). During the first 2–3 days, 10 uM Y-27632 was added to the culture medium to prevent anoikis. After reaching a size greater than 200 µm, organoids were passaged by pipetting using the Gentle Cell Dissociation Reagent (STEMCELL Technologies, Vancouver, BC, Canada) according to the manufacturer’s instructions. Images were acquired using an IX73 inverted microscope (Olympus Corporation, Tokyo, Japan; magnification, ×40) and an EVOS FL Cell Imaging system fluorescence microscope (Thermo Fisher Scientific, Inc., Walthan, MA, USA; magnification, ×200). 

### 2.4. Radiation Response Assays

For survival fraction analysis, patient-derived organoids were resuspended in TrypLE Express (Thermo Fisher Scientific, Inc.). Over subsequent days, organoids were treated with ionizing radiation, delivered by a 137Cs γ-ray source (Atomic Energy of Canada Ltd., Chalk River, Ontario, Canada) at a dose rate of 3.81 Gy/min. After 14 days, organoids were analyzed using a neo Cell3Imager (Screen) with organoid optimized analysis recipes (organoid diameter min 93, max 2907; organoid area min 6833, max 6640106; circularity min 0.24, max 1). For viability assays, organoids were distributed into wells of a 96-well plate. Over following days, organoids were treated with 0, 2, 4 and 6 Gy and their viability was evaluated using a CellTiter 96 AQUEOUS One Solution (Promega, Madison, WI, USA) after 7 days according to the manufacturer’s instructions. Optical density was measured on a BioTek Eon microplate absorbance reader (BioTek Instruments Inc., Winooski, VT, USA) at 490 nm. For proliferation assay, organoids were incubated with 10 µM EdU for 2 h and evaluated using Click-iT Plus EdU Imaging kits (Thermo Fisher Scientific, Inc.) according to the manufacturer’s instruction. After radiation, organoid images were acquired using an EVOS FL Cell Imaging System (Thermo Fisher Scientific, Inc.). For all analysis and experiments, the passage number of cultured organoids was between 10 and 30.

### 2.5. RNA Isolation

Total RNA was isolated using QIAzol reagent (Qiagen, Hilden, Germany) from irradiation naive organoids. RNA quality (expressed as RNA integrity number) was assessed on an Agilent 2100 bioanalyzer using an RNA 6000 Nano Chip (Agilent Technologies, Amstelveen, The Netherlands). RNA was quantified using a NanoDrop 2000 spectrophotometer (ND-2000; Thermo Fisher Scientific, Inc.).

### 2.6. RNA-seq

RNA sequencing (RNA-seq) was performed on high-quality RNA samples (RNA integrity number > 7) from the six patient-derived organoids, RR1–3 and RS1–3. The six separate samples were multiplexed into each lane and sequenced on a HiSeq 4000 system (Illumina, San Diego, CA, USA). The sequenced libraries were aligned to the human genome (hg19) reference sequence using HISAT v2.1.0 [18]. The reference genome sequence and its annotation were downloaded from the UCSC genome browser (https://genome.ucsc.edu/ accessed on 1 April 2020).

### 2.7. Identification of Differentially Expressed Genes and Data Analysis

QuantSeq 3′ mRNA-Seq reads were aligned using Bowtie2 [19]. Bowtie2 indices were generated from the genome assembly sequence or the representative transcript sequences for alignment to the genome and transcriptome. The alignment file was used for assembling transcripts, estimating their abundances, and detecting differentially expressed genes (DEGs). DEGs were determined based on counts from unique and multiple alignments using coverage in Bedtools [20]. Read Count data were processed based on a quantile normalization method using edgeR, a Bioconductor software package [21]. Data mining and graphic visualization were performed using Excel-based Differentially Expressed Gene Analysis (ExDEGA; Ebiogen Inc., Seoul, Korea). Probe sets without corresponding gene symbols were removed. In this study, differences with a *P*-value < 0.05 and absolute log_2_ (fold change) ≥ 1 were considered statistically significant. 

### 2.8. Gene and Pathway Enrichment Analyses of DEGs

The Database for Annotation, Visualization and Integrated Discovery (DAVID; https://david.ncifcrf.gov/ accessed on 11 November 2020), an online biological information database, was used for pathway enrichment analysis of DEGs. The Gene Ontology (GO) term enrichment analysis annotated by the DAVID database is composed of three attributes: molecular function (MF), biological process (BP), and cellular component (CC). Pathway enrichment analyses were conducted using the Kyoto Encyclopedia of Genes and Genomes (KEGG) and REACTOME, which are tools of the DAVID website. *P*-values < 0.05 were considered statistically significant. 

### 2.9. Protein–Protein Network and Module Analysis

In this study, protein–protein (PPI) networks were mapped using Cytoscape (version 3.8.2; https://cytoscape.org/ accessed on 11 November 2020), a public-access software that can graphically edit, display, and analyze the network. Significant modules in PPI networks were identified using Molecular Complex Detection (MCODE), a plug-in app of Cytoscape designed to analyze densely connected regions by clustering a given network. Hub genes were identified using the cytoHubba analysis in Cytoscape. Analyses with ClueGO, a Cytoscape plug-in, were performed using databases updated in May 2020. 

### 2.10. Quantitative Reverse Transcription-Polymerase Chain Reaction (qRT-PCR)

Total RNA was extracted using QIAzol reagent (Qiagen), then reverse transcribed into cDNA using amfiRivert reverse transcriptase (GenDEPOT, Katy, TX, USA) according to the manufacturer’s instructions. cDNA was amplified by PCR on a Mic Real-Time PCR system (Bio Molecular Systems, Upper Coomera, QLD, Australia) using Luna Universal qPCR master mix (New England Biolabs Inc., Ipswich, MA, USA) and primer pairs specific for target genes (Table S1). Results were analyzed using the ΔCT and 2^−ΔΔCT^ quantification method. 

### 2.11. Validation of Genetic Alterations in Candidate Genes

Genetic alterations in candidate genes in the colorectal adenocarcinoma dataset were analyzed using cBioPortal (http://cbioportal.org/ accessed on 11 November 2020), an online analysis platform for multidimensional cancer genomic data that provides collective visualization of genes, samples, and data types. 

### 2.12. DNA Extraction and Sodium-Bisulfite Modification

Genomic DNA (gDNA) was extracted from patient-derived organoids using QIAamp DNA Mini Kit (Qiagen). The bisulfite reaction was carried out with 2 μg gDNA in a reaction volume adjusted to 50 μL with sterile water, to which 130 μL of the provided CT conversion reagent was added. Thereafter, sample tubes were placed in a thermal cycler (MJ Research, Waltham, MA, USA) and incubated first for 15 min at 3 °C and then for 16 h at 50 °C, then stored at 4 °C. The resulting bisulfite-modified DNA was purified using an EZ DNA Methylation Kit (Zymo Research, Orange, CA, USA) and eluted with 40 μL of the provided M-Elution buffer. Finally, a 1-μL aliquot was used as a template for subsequent PCR.

### 2.13. Pyrosequencing Analysis

Pyrosequencing primers (one biotinylated and one non-biotinylated) were designed to amplify 1 to 6 CpG dinucleotides in each target-gene promoter. PCR was carried out in a volume of 25 μL containing 10 ng of converted gDNA, PyroMark PCR Kit master mix (Qiagen), 1 μL of 10 pmol/μL nonbiotinylated primer, and 1 μL of 10 pmol/μL biotinylated primer. The primers utilized (Table S2) were designed using PSQ Assay Design software (Qiagen). Amplification was carried out according to the general guidelines suggested in the pyrosequencing protocol as follows: denaturing at 95 °C for 5 min, followed by 45 cycles of 95 °C for 30 s, 53 °C for 30 s and 72 °C for 30 s, with a final extension at 72 °C for 10 min. The quality and purity of PCR products (10 μL aliquots) were confirmed by 2% agarose gel electrophoresis and staining with TopRed Nucleic Acid Gel stain (BioPure, Horndean, UK). Pyrosequencing was performed on a PyroMark ID system using PyroMark Q48 magnetic beads (Qiagen) and PyroMark Q48 Advanced CpG Reagents (Qiagen), as described by the manufacturer without further optimization. The methylation percentage was calculated as the average degree of CpG site methylation. All pyrosequencing experiments were performed three times.

### 2.14. Statistical Analysis and Graphical Representation

Statistical analyses were performed using GraphPad Prism 7 (GraphPad Software, San Diego, CA, USA) or Microsoft Excel 2010 (Microsoft Corp., Redmond, WA, USA). Data obtained from at least three independent experiments are presented as means ± standard deviation (SD). The statistical significance of differences between two means was analyzed by Student’s *t*-test, and a *P*-value < 0.05 was considered significant.

## 3. Results

### 3.1. Classification of LARC Patients Based on Clinical Findings 

Six pathologically proven LARC patients who visited Korea cancer center hospital in Seoul (South Korea) were selected and asked to provide written consent for collection of biopsy samples and clinical images. The patients were assessed by pelvis magnetic resonance imaging (MRI) and 18-fluoro-2-deoxy-glucose positron emission tomography (PET) scanning before and after NCRT. After standard NCRT, all patients received radical surgeries. Patient clinical information is listed in Table 1. 

**Figure 1:**
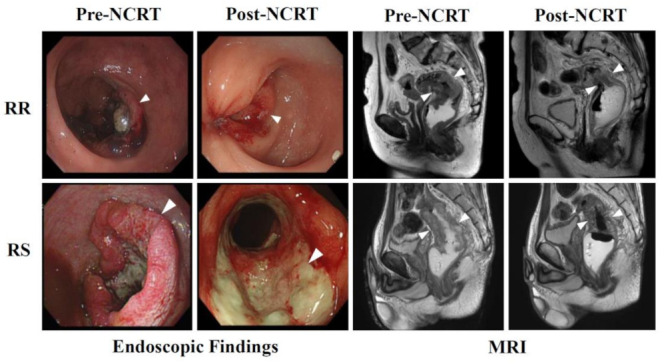
NCRTs cause differential effects in LARC patients. Endoscopic findings (left panels) and magnetic resonance images (right panels) are shown for radioresistant (RR; upper panels) and radiosensitive (RS; lower panels) patients. Pre- and Post-NCRT images are compared. White arrows indicate LARC tissues.

Individual patients were clinically diagnosed before and after NCRTs. All patients showed some degree of lymph node invasion before NCRTs. Patients were categorized into two groups—complete response (n = 3) and poor response (n = 3)—based on the tumor regression grade (TRG) of NCRT. Representative endoscopic images and pelvic MRIs for radio-resistant (RR) and radio-sensitive (RS) patients are shown in Figure 1. Although post-surgical specimens from RS patients were found to be TRG 0 (i.e., pCR), images of RS patients revealed only minimal dissipation of LARC after NCRT (Figure 1, Table 1). These cases strengthen the concept that preoperative clinical findings may be insufficient for discriminating the radio-responsiveness of LARC, increasing the demand for developing biomarkers associated with radio-responsiveness.

### 3.2. Establishment of Patient-Derived Organoids from LARC Patients

**Figure 2:**
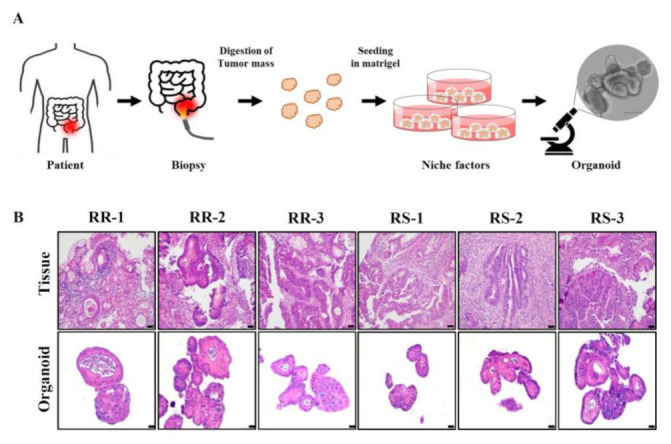
Establishment of LARC patient-derived organoids. (**A**) A scheme for preparation of patient-derived organoids is shown. (**B**) Patient-derived organoids and the corresponding primary LARCs are compared using H&E staining. Scale bar: 20 µm.

In an effort to ensure the consistency of specimens and minimize contamination by non-cancer cells during sample preparation, we adopted an organoid system—a proven model system for investigating gastrointestinal tissues [15]. Organoids were established from biopsy samples collected from LARC patients as depicted in Figure 2A. Briefly, collected biopsy samples were treated with digestive enzymes to dissociate cells. Single cells were then seeded and incubated in Matrigel containing niche factors. Patient-derived organoids showed a similar morphology that mimicked the original tissue architecture (Figure 2B). Also, individual organoids were viable (Figure S1) and proliferated at a similar rate (Figure S2).

**Figure 3:**
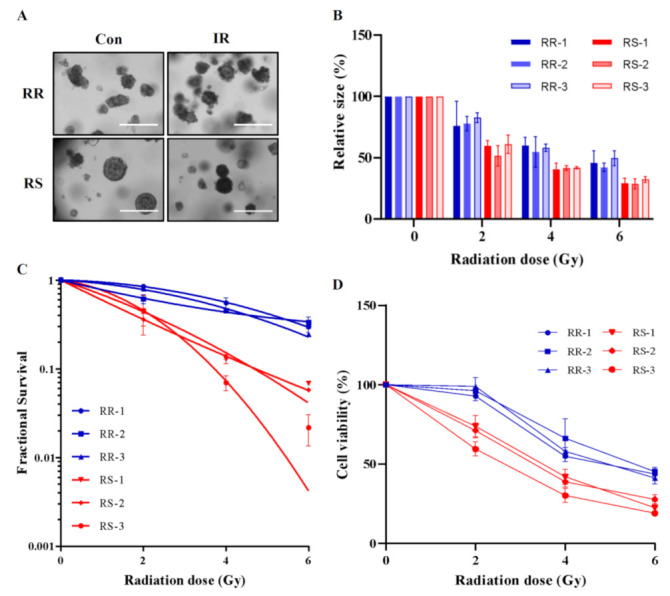
Patient-derived organoids show differential radio-responsiveness. (**A**) Brightfield images of RR and RS organoids after 0 Gy and 5 Gy irradiation are shown. Scale bar: 400 μm (**B**) Changes in the size of the six organoids (RS1–3 and RR1–3) after 0, 2, 4, and 6 Gy irradiation are shown (n = 4 independent experiments). (**C**) Radiation dose-responses are measured based on survival fraction curves of patient-derived organoids (n = 4 independent experiments). (**D**) The viability of patient-derived organoids after 0, 2, 4, and 6 Gy irradiation (n = 6 independent experiments) was assessed by MTS assay. Differential expression is considered significant at *P* < 0.01. Data are normalized to those of control organoids and are presented as mean ± SD.

Next, we assessed the radio-sensitivity of organoids. Microscopic observations showed that γ-irradiation markedly decreased the size of RS patient-derived organoids, but not the size of RR patient-derived organoids (Figure 3A). The effects of γ-irradiation were confirmed based on multiple assessments, including organoid size (Figure 3B), number of organoids (Figure 3C), EdU incorporation (Figure S2) and viability, determined by MTS assay (Figure 3D). Irradiation-induced inhibition of RS organoids was ~2-fold greater than that of RR organoids, consistent with the clinical data.

### 3.3. Selection of Differentially Expressed Genes Using RNA-seq of Organoids

**Figure 4:**
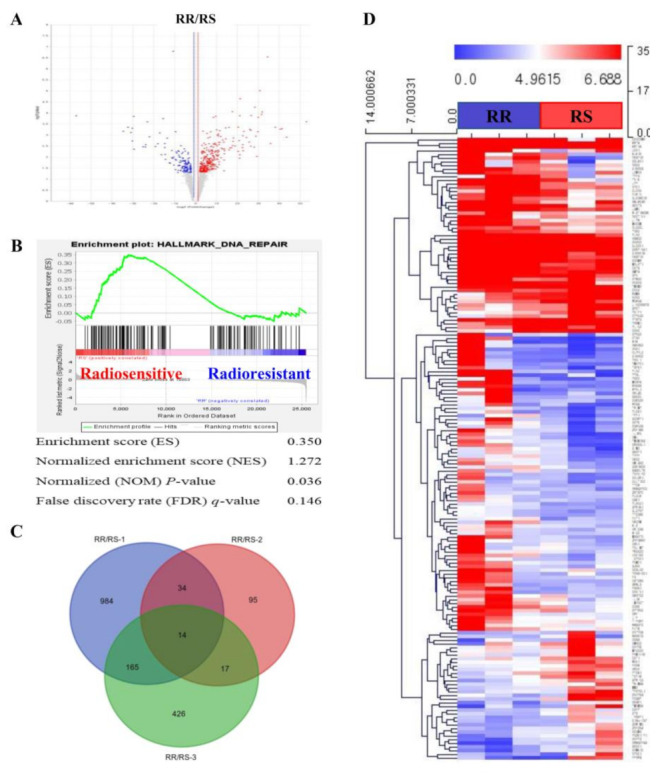
Analysis of DEGs in patient-derived organoids. (**A**) Significant DEGs between RR and RS organoids, in the form of log_10_ (*P*-value) versus log_2_ (fold change), are presented graphically as volcano plots. (**B**) Enrichment plots for GSEA, processed using the expression difference-ranked gene list, shows enrichment of the DNA repair-related gene set. (**C**) Venn diagram shows the number of differentially expressed genes between RS and RR organoids. (**D**) Heatmap illustrates significant DEGs between RR (blue bar) and RS (red bar) organoids. Representations of genes were processed using the general linear model likelihood ratio test (*P* < 0.05 and absolute log_2_ fold change >1).

To identify genes associated with the radio-responsiveness of patient-derived organoids, we analyzed candidate genes that were differentially expressed between RR and RS organoids. Gene expression profiles were generated by RNA-seq. A total of 27,685 genes were analyzed, 1741 of which satisfied our criteria for differentially expressed genes (DEGs) (|FC| ≥ 2 and Raw. *P* < 0.05) (Figure 4A). We then performed gene set enrichment analysis (GSEA) [22] on these selected DEGs (Table 2). This analysis indicated the involvement of protein-, RNA- and DNA-maintenance pathways. Interestingly, cell-cycle and DNA-repair pathways were also among those associated with DEGs (Table 2, Figure 4B), providing insight into the mechanism underlying radio-resistance in LARC.

A Venn diagram of the 1741 DEGs indicated significant variations among patient-derived organoids, with RS-1, RS-2 and RS-3 samples exhibiting 1197, 160 and 622 DEGs, respectively (Figure 4C). A total of 231 genes that sorted to intersections of the Venn diagram common for at least two RS organoids were used for subsequent bioinformatic analysis. A heatmap analysis [23] confirmed the radio-responsiveness categories (Figure 4D), ensuring the consistency of our dataset between patients and organoids. A gene ontology (GO) analysis [24] of significantly enriched genes was also performed to obtain overview information about the function of protein products of our DEGs (Table 3). Cell cycle and proliferation pathways (biological process), extracellular and plasma membrane (cellular compartment), and receptors and scaffolding proteins (molecular function) were the GO properties most frequently associated with DEGs.

### 3.4. Functional Classification of DEGs Associated with Radio-Responsiveness

**Figure 5:**
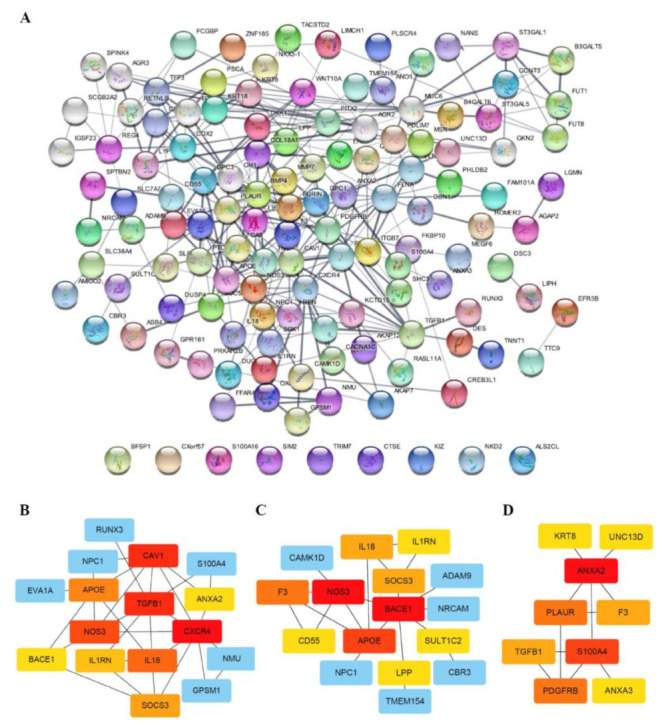
PPI network and sub-networks generated from DEGs. (**A**) The PPI network was processed using the STRING plug-in of the Cytoscape program (version 3.8.2). Each circle represents a gene (node), and connections between circles (edges) represent direct or indirect interactions. Of the 231 genes that were differentially expressed between RR and RS organoids, 130 were functionally linked with 289 edges. PPI enrichment *P*-values < 0.04 were considered significant. (**B**–**D**) Module analyses. Module clusters were extracted using MCODE and cytoHubba analyses. Hub genes are indicated in red, and co-expressed genes are indicated in orange, yellow or blue according to their degree of importance. Module 1 (**B**) contains 16 nodes and 30 edges. Module 2 (**C**) contains 16 nodes and 20 edges. Module 3 (**D**) contains 9 nodes and 11 edges.

Protein–protein interaction (PPI) networks can reveal physical contacts between protein pairs and provide small subsets of biological pathways [25]. Using this approach, we generated a PPI network containing 130 nodes and 289 edges from 231 DEGs (Figure 5A, Table S3). The resulting network was still very complex; thus, we subsequently performed a minimal common oncology data elements (MCODE) analysis with specific modules [26]. The networks were also confirmed by cytoHubba analysis [27], which generated similar hub networks (Figure 5B–D). This subnetwork analysis identified tumor growth factor (TGF)-β, BACE1 (β-secretase 1), APOE (apolipoprotein E) and ANXA2 (annexin A2), among others, as hub genes. 

**Figure 6:**
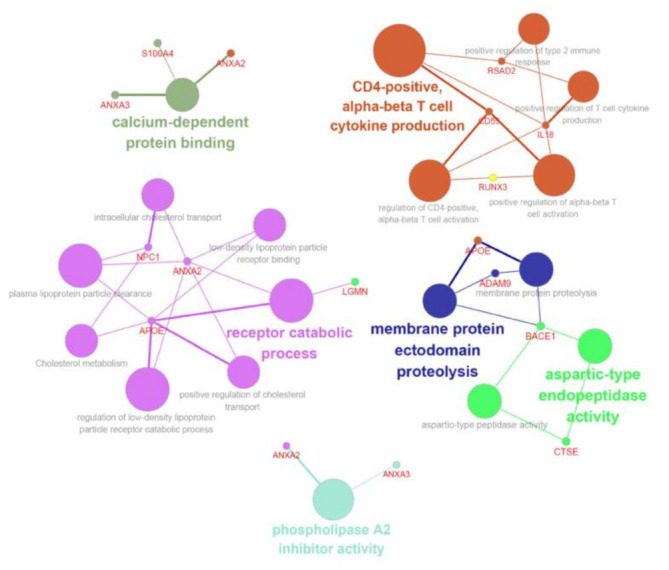
ClueGo analysis-based enrichment maps derived from GO terms associated with DEGs. Highly interconnected GO terms are presented. Terms in bold font indicate top GO terms. Gene names within subgroups were generated using ClueGO default settings. All GO terms shown are statistically significant (*P* < 0.05 with Bonferroni correction).

Biological interpretation can be improved by organizing separate GO/pathway term networks using ClueGo and module analysis [28]. This analysis revealed various additional processes linked to radio-responsiveness, including calcium-dependent interactions, immune cell activation, receptor catabolic processes, and plasma membrane proteolysis (Figure 6). 

### 3.5. Validation of Candidates from RNA-seq Analysis

**Figure 7:**
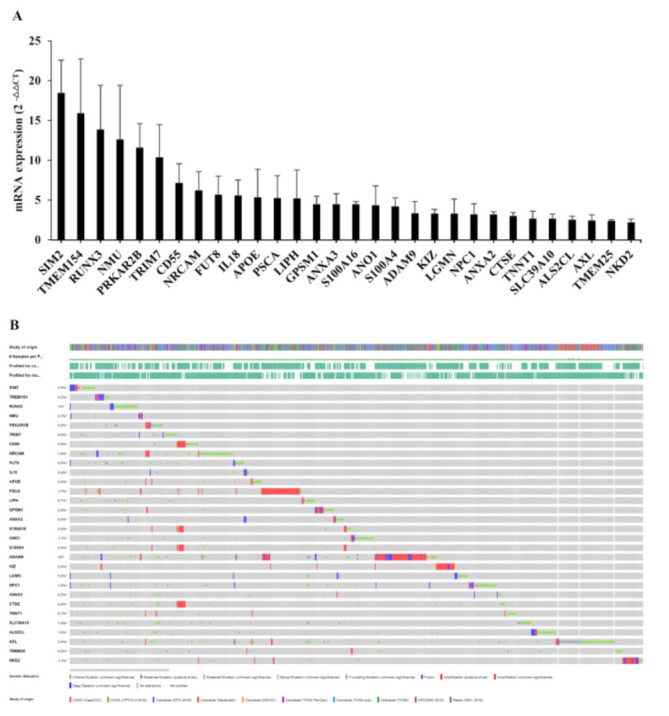
Verification of candidate genes by qRT-PCR and cBioPortal analysis. (**A**) The graph depicts mRNA levels of candidate genes that are differentially expressed between RR and RS organoids. All candidate genes were significantly upregulated in RR organoids. Differential expression is considered significant at *P* < 0.05. Error bars indicate standard deviations (n = 3). (**B**) Candidate genes were queried for genetic alterations in colorectal adenocarcinoma datasets (http://cbioportal.org/ accessed on 11 November 2020). Alterations were found in 0.3% to 3% of the respective analyses and are depicted graphically. Many candidate genes exhibited amplification alterations.

We next verified gene expression profiles using quantitative RT-PCR analysis, selecting the top 30 genes with the highest fold-changes for validation. The expression data were normalized to the geometric mean of the housekeeping gene, GAPDH, to control the variability in expression levels and analyzed using the 2^−ΔΔCT^ method. These top genes included those encoding proteins involved in calcium-dependent interactions (ANXA2, S100A4…), immune cell activation (CD55, IL18, RUNX3…), receptor catabolic processes (NPC1, APOE, LGMN…), and plasma membrane proteolysis (ADAM9, BACE1, CTSE…). The molecular properties of these gene products are summarized in Supplementary Data (Table S4). As shown in Figure 7A, qRT-PCR analyses confirmed upregulation of our candidates in RR organoids. 

Using the cBioPortal for cancer genomics, originally developed for interactive exploration of multidimensional cancer genomic datasets [29], we extracted a dataset from the clinical expression dataset of colorectal adenocarcinoma containing 10 studies and 3953 patient samples (Figure 7B). The majority of our candidates were found in the extracted dataset, consolidating the credibility of our candidate list. 

### 3.6. CTSE Is Epigenetically Regulated

The expression of our candidate DEGs was strongly skewed toward enhancement in RR organoids (Figure 7A). Upregulation of genes can be achieved through genetic regulation involving transcription factors, or epigenetically through demethylases or acetylases. Notably, it has been found that aberrant DNA methylation is involved in colorectal cancer progression; specifically, an 8% to 10% reduction in 5-methylcytosine content has been observed in colorectal cancers compared with normal tissues [30].

**Figure 8:**
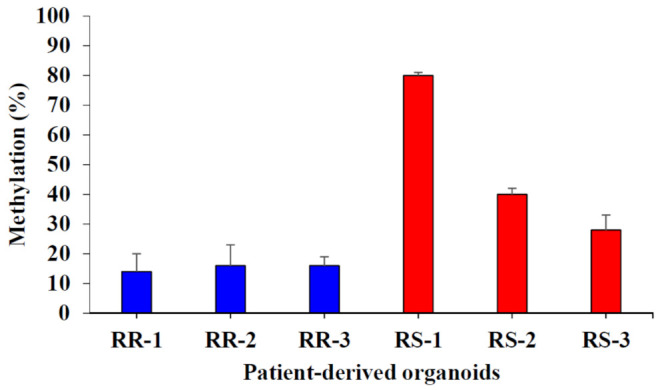
Distinct DNA methylation profiles of the *CTSE* gene are correlated with the radio-responsiveness of patient-derived organoids. Methylation levels of the *CTSE* gene in RR and RS organoids were assessed by bisulfite sequencing. Differential expression is considered significant at *P* < 0.05. Error bars indicate standard deviations (n = 3).

Among the genes on our candidate list, *CTSE*, encoding cathepsin E, is known to be regulated by DNA methylation [31]. Therefore, we hypothesized that a reduction in DNA methylation might contribute to the radio-responsiveness of patient-derived organoids. An assessment of DNA methylation between RS and RR organoids showed that RS organoids exhibited a higher methylation pattern in the *CTSE* gene than RR organoids (Figure 8), possibly explaining enhanced expression of *CTSE* in RR organoids. These data further suggest that CTSE protein levels are regulated by DNA methylation status, which could thus be used as a biomarker for radio-responsiveness.

## 4. Discussion

The current standard of care for LARC is to apply the same treatments to all patients regardless of their response to NCRTs. Such a uniform treatment approach inevitably leads to over or under treatment for some LARC patients. Achieving the goal of personalized medicine requires categorization of patients as a first step toward successful treatment. In the case of CRC, an international consortium formed to simplify classifications based on the gene-expression profiles of patients [32] suggested four consensus molecular subtypes (CMS) of CRC. This effort has helped clarify the patterns of CRC, enabling physicians to apply treatment options based on the resulting categorizations. For LARC specifically, improving standard treatments requires new biomarkers that discriminate radio-responsiveness. In the last decade, numerous studies seeking to establish routine prediction of radio-responsiveness have reported specific biomarkers indicative of response to NCRT [33], including clinical features, PET/CT imaging and blood cell ratios, among others, but these efforts have met with limited success. Detailed molecular approaches, such as genetic mutation and metabolite analyses, have yielded promising results, and a combined approach that simultaneously analyzes multiple biomarkers using RNA arrays has become increasingly popular. Although no biomarkers have been clinically proven, genes involved in regulating the cell cycle, apoptosis, hypoxia, cancer progression and/or DNA repair have shown some promise as predictors of pCR [33].

In this report, we adopted an organoid model system to identify genes associated with radio-responsiveness. DEGs associated with radio-responsiveness were determined by biological and bioinformatic analysis of gene expression profiles of LARC patient-derived organoids. On the basis of this expression profiling, we selected 30 candidate DEGs for further analysis, including transcription factors (SIM2, RUNX3…), transmembrane proteins (CD55, TMEM154, NRCAM…), secreted proteins (IL18, ADAM9, SLC39…), and signal transducers (GPSM1, ALS2CL, PRKAR2B…). The enriched pathways associated with these genes suggested that radio-resistance requires robust DNA repair processes, enhanced immune responses and/or diminished or absent cell cycle arrest activity. These identified pathways overlap with previously reported pathways; however, the individual genes are quite different— differences that could be attributable to patient- or organoid-specific effects. 

We next looked for possible regulatory mechanisms governing expression of these genes. Feinberg et al. previously reported that a reduction in DNA methylation is involved in CRC progression [30]. Such global hypo-methylation of LARC DNA could explain our expression data, in which DEGs were uniformly skewed towards upregulation in RR organoids (Figure 7A). To test this idea, we focused on the *CTSE* gene, encoding an aspartic protease known to function in protein turnover, antigen presentation, and apoptosis [34]. CTSE is mainly expressed in the plasma membrane of immune and gastrointestinal cells. Notably, CTSE upregulation has been linked to multiple cancers, and less differentiated tumors tend to show higher expression of CTSE [35]. Consistent with a recent report by Hiramatsu et al. that hypo-methylation can regulate *CTSE* expression [31], we found that DNA methylation levels at the *CTSE* gene are lower in radio-resistant organoids than in radio-sensitive organoids (Figure 8). Taken together with this previous report, our findings suggest that DNA methylation can regulate the expression of a DEG—specifically *CTSE*—that is linked to the radio-responsiveness of LARC. 

## 5. Conclusions

Additional studies with larger populations will be required to validate the effectiveness of these markers in predicting patient outcomes. Such future large-scale clinical studies could employ custom-made microarray chips, in which our candidate sequences could be hybridized to a glass slide together with quality control sequences. Measuring DNA methylation at specific sites could further enhance the sensitivity and specificity of radio-responsiveness detection. Our hope is that all effort will be made to apply insights regarding tailored application of radiation therapy and its effects in LARC, initiating a new era of personalized medicine.

## Figures and Tables

**Table 1:** Clinical Characteristics of LARC Patients.

Sample No.	RR-1	RR-2	RR-3	RS-1	RS-2	RS-3
Sex	Male	Male	Female	Male	Male	Female
Age (years)	77	71	76	49	64	62
BMI (Kg/m^2^)	16.5	22.7	24.3	17.7	24.6	17.3
Clinical stage	T3N+	T3N+	T3N+	T3N+	T2N+	T3N+
Tumor length, preRT MRI (cm)	4.2	5.4	6.5	6.5	3.8	3.5
Tumor length, postRT MRI (cm)	2.3	5.3	2.5	4	1.6	2.5
Tumor length, pathology (cm)	2.5	7.5	3.5	3.8	1	1.2
Regression grade	Minimal	Minimal	Minimal	Complete	Complete	Complete
ypStage	T3N1M0	T3N1M0	T3N0M0	T0N0M0	T0N0M0	T0N0M0
Recurrence	Lung metastasis	Local recurrence	No	No	No	No
Follow up after surgery (month)	19	17	18	27	23	24
Status at last visit	Dead due to lung- metastasis	Alive with recurrence	Alive without recurrence	Alive without recurrence	Alive without recurrence	Alive without recurrence

Abbreviations: BMI, body mass index; RT, radiotherapy; MRI, magnetic resonance image; ypStage, pathologic stage after neoadjuvant treatment at surgical specimen.

**Table 2:** Gene Set Enrichment Analysis of Radiation Response-related DEGs.

Gene Sets Details	SIZE	ES	NES	NOM *P*-Value	FDR *q*-Value
KEGG_PROTEASOME	44	0.51	1.71	0.000	0.073
KEGG_CELL_CYCLE	125	0.48	1.61	0.000	0.104
KEGG_SPLICEOSOME	126	0.50	1.53	0.000	0.203
KEGG_RNA_POLYMERASE	29	0.59	1.52	0.000	0.198
KEGG_PYRIMIDINE_METABOLISM	98	0.46	1.49	0.000	0.242
KEGG_MISMATCH_REPAIR	23	0.60	1.49	0.000	0.209
KEGG_BASE_EXCISION_REPAIR	33	0.53	1.49	0.000	0.185

Abbreviations: ES, enrichment score; NES, normalized enrichment score; NOM *P*-value, normalized *P*-value; FDR, false discovery rate.

**Table 3:** GO Analysis of Radiation Response-related DEGs.

Category	Term	Description	Count	*P*-Value
BP	GO:0050680	Negative regulation of epithelial cell proliferation	8	4 × 10^−5^
	GO:0045786	Negative regulation of cell cycle	6	4 × 10^−4^
	GO:0001525	Angiogenesis	11	5 × 10^−3^
	GO:0001934	Positive regulation of protein phosphorylation	8	6 × 10^−3^
	GO:0001937	Negative regulation of endothelial cell proliferation	4	1 × 10^−2^
	GO:0098609	Cell-cell adhesion	11	2 × 10^−2^
	GO:0007179	Transforming growth factor beta receptor signaling pathway	6	2 × 10^−2^
	GO:0042632	Cholesterol homeostasis	5	2 × 10^−2^
	GO:0051044	Positive regulation of membrane protein ectodomain proteolysis	3	3 × 10^−2^
	GO:0016477	Cell migration	8	3 × 10^−2^
CC	GO:0070062	Extracellular exosome	79	2 × 10^−6^
	GO:0005886	Plasma membrane	98	1 × 10^−4^
	GO:0005615	Extracellular space	42	1 × 10^−4^
	GO:0000139	Golgi membrane	22	1 × 10^−3^
	GO:0045121	Membrane raft	11	2 × 10^−3^
	GO:0031225	Anchored component of membrane	8	3 × 10^−3^
	GO:0005913	Cell-cell adherens junction	13	8 × 10^−3^
	GO:0005737	Cytoplasm	107	1 × 10^−2^
	GO:0048471	Perinuclear region of cytoplasm	19	2 × 10^−2^
	GO:0005576	Extracellular region	38	3 × 10^−2^
**MF**	GO:0001618	Virus receptor activity	6	7 × 10^−3^
	GO:0097110	Scaffold protein binding	5	9 × 10^−3^
	GO:0005509	Calcium ion binding	22	1 × 10^−2^
	GO:0004861	Cyclin-dependent protein serine/threonine kinase inhibitor activity	3	1 × 10^−2^
	GO:0004859	Phospholipase inhibitor activity	3	1 × 10^−2^
	GO:0098641	Cadherin binding involved in cell-cell adhesion	11	3 × 10^−2^
	GO:0015485	Cholesterol binding	4	3 × 10^−2^
	GO:0004872	Receptor activity	9	3 × 10^−2^
	GO:0005528	FK506 binding	3	5 × 10^−2^
	GO:0030506	Ankyrin binding	3	5 × 10^−2^

Abbreviations: BP, biological process; CC, cytosolic compartment; MF, molecular function.

## Data Availability

Not applicable.

## Supplementary Materials

The following are available online at https://www.mdpi.com/article/10.3390/biology10060500/s1, Figure S1: Ki-67 fluorescence microsopic images of patient-derived organoids. Figure S2: EdU incorporations of patient-derived organoids were measured upon γ-irradiation. Table S1: qRT-PCR primer sequences for the verification of candidates. Table S2: Gene pyrosequencing conditions and primers. Table S3: 213 genes differentially expressed between RR and RS organoids. Table S4: Functional annotations of candidate genes.
