# Supplementary material for: Gene Expression Profiles Associated with Radio-Responsiveness in Locally Advanced Rectal Cancer
Source: Biology (Basel). 2021 Jun 3;10(6):500. doi: 10.3390/biology10060500 (PMC8226560; doi:10.3390/biology10060500)
Supplement: Supplementary file 1 [file biology-10-00500-s001.zip › Supplemental Figures.pdf]

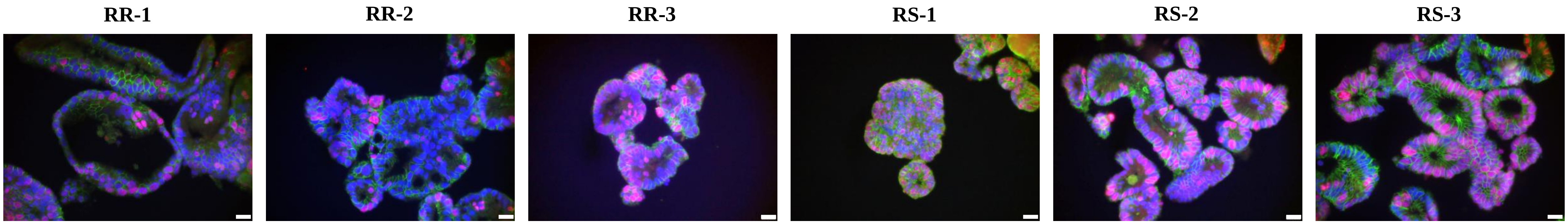

Figure S1. Ki-67 fluorescence microscopic images of patient-derived organoids.
Patient-derived organoids (RRs and RSs) were stained with DAPI (blue), E-cadherin (green) and Ki-67 (red). Fluorescence microscopy images were acquired using EVOS EL cell imaging system. Scale bar: 100 μm.

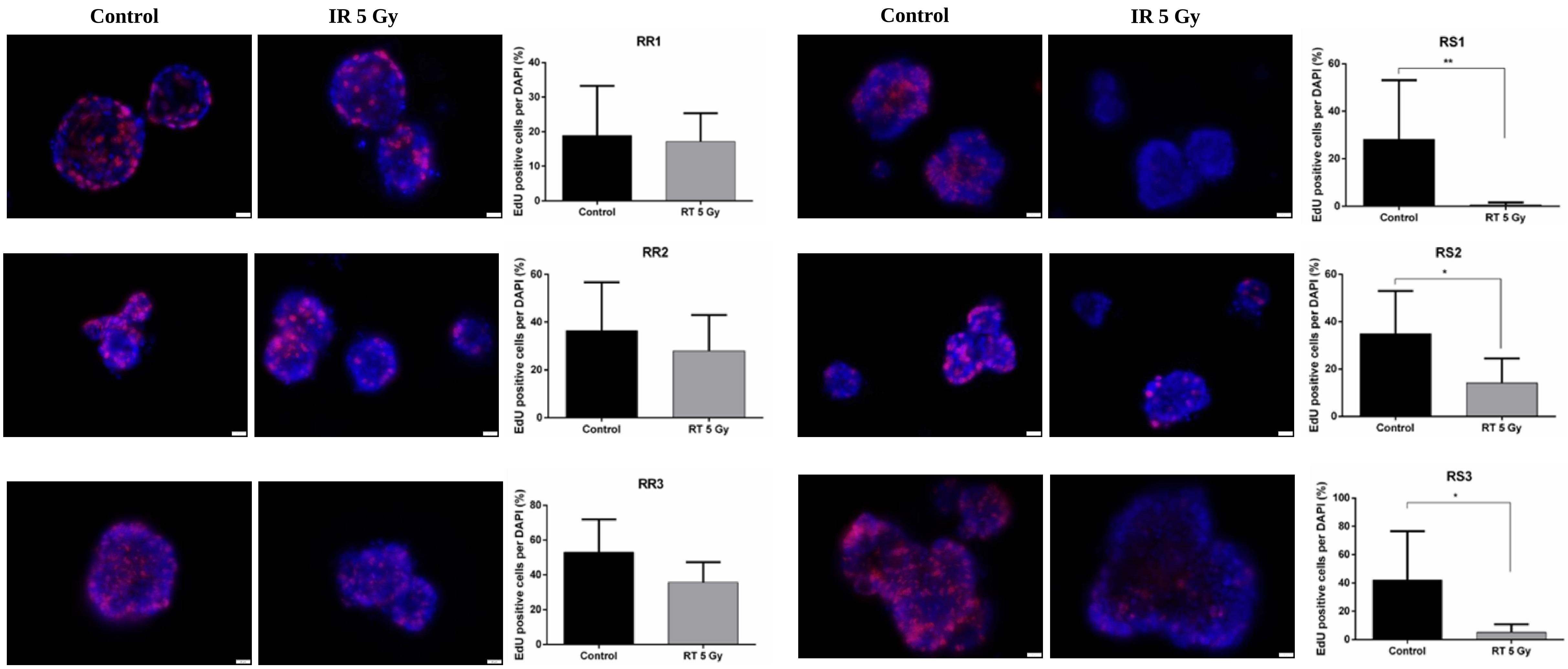

Figure S2. EdU incorporations of patient-derived organoids were measured upon γ-irradiation.
(left) Fluorescence microscopic images of EdU incorporation in RR-1, 2, 3 and RS-1, 2, 3 with or without **γ-**irradiation. Scale bar: 20 μm. Blue, DAPI; red, EdU. (right) statistical analysis representing EdU-positive cells per DAPI-stained cells (n=3). *P<0.01, * *P<0.001.
