# Supplementary material for: Gene Expression Profiles Associated with Radio-Responsiveness in Locally Advanced Rectal Cancer
Source: Biology (Basel). 2021 Jun 3;10(6):500. doi: 10.3390/biology10060500 (PMC8226560; doi:10.3390/biology10060500)
Supplement: Supplementary file 1 [file biology-10-00500-s001.zip › Supplemental Table S1. primer.pdf]

Table S1. qRT-PCR primer sequences for the verification of candidates.

Gene Name		Primer Sequences	Accession Number	Product (bp)
ADAM9	(F)	CTTGCTGCGAAGGAAGTACCTG	NM_003816	121
	(R)	CACTCACTGGTTTTTCCTCGGC		
ALS2CL	(F)	TGAGCGCTACATTGGCATGTGG	NM_001190707	152
	(R)	CCTCATACAGGGAGTCGTCTTC		
ANO1	(F)	GAAGCGGAAACAGATGCGACTC	NM_018043.6	102
	(R)	CTGGCTTCGTATTCAGCTCTAGG		
ANXA2	(F)	TCGGACACATCTGGTGACTTCC	NM_004039	135
	(R)	CCTCTTCACTCCAGCGTCATAG		
ANXA3	(F)	CTCCACCAGCAGTCTTTGATGC	NM_005139	114
	(R)	CCTTCATTTGCCTGCTTGTCCTG		
APOE	(F)	GGGTCGCTTTTGGGATTACCTG	NM_000041.4	124
	(R)	CAACTCCTTCATGGTCTCGTCC		
AXL	(F)	GTTTGGAGCTGTGATGGAAGGC	NM_021913.5	121
	(R)	CGCTTCACTCAGGAAATCCTCC		
CD55	(F)	CACGGAGTACACCTGTTTCCAG	NM_000574.5	134
	(R)	CCCAAGCAAACCTGTCAACGTG		
CTSE	(F)	TCATTGGAGCCGACCAAGTC	NM_001317331.2	198
	(R)	GCAAGTCCACCAGGTTCTGA		
FUT8	(F)	GACAGAACTGGTTCAGCGGAGA	NM_004480	130
	(R)	GCAGTAGACCACATGATGGAGC		
GPSM1	(F)	AGGCGCCTCTACTCCAGGAT	NM_001145638.3	233
	(R)	AGCAGGAGGTCATGCTTGTG		
IL18	(F)	GATAGCCAGCCTAGAGGTATGG	NM_001243211.2	121
	(R)	CCTTGATGTTATCAGGAGGATTCA		
KIZ	(F)	GGTCAGCATGTTGCCACCTTGA	NM_001163022.3	113
	(R)	CTGTGAATAGCTGACCTACGGC		
LGMN	(F)	CCTGAAGATGGAGGCAAGCACT	NM_005606	127
	(R)	GTTCGTCAGGAATCCCATTGCG		
LIPH	(F)	CAACGGGAAACCTCACCAAGAC	NM_139248.3	146
	(R)	AGCCAGGTTGATCCAATCCTCC		
NKD2	(F)	GACAACTCCTCAGCGCAGATGA	NM_033120.4	152
	(R)	GTCATAGAGCGTGAACGTCCAC		
NMU	(F)	AGCTCGTTCCTCACCTGCATGA	NM_006681.4	129
	(R)	CTGCTGACCTTCTTCCATTCCG		
NPC1	(F)	TCTCTTTGCGGGATTGGCAGTC	NM_000271	146
	(R)	CGCTTGTTCCATCTTCAGCACC		
NRCAM	(F)	TGTGGCTGAAGGACAACAGGGA	NM_001037132.4	138
	(R)	AGACGCTGTCCAGAGTGGTGTT		

PRKAR2B	(F)	AACCGATTCACAAGGCGTGCCT	NM_002736.3	144
	(R)	CAGCAGGATGTCTTTGCAAGCC		
PSCA	(F)	TGCTGTGCTACTCCTGCAAAGC	NM_005672.5	160
	(R)	GAGTCATCCACGCAGTTCAAGC		
RUNX3	(F)	GGCAATGACGAGAACTACTCCG	NM_004350.3	129
	(R)	GATGGTCAGGGTGAAACTCTTCC		
SIM2	(F)	TGTCTTGGCGAAAAGGAACGCG	NM_005069.6	128
	(R)	CCACAATCTGGTAGCAGGAGTC		
SLC39A10	(F)	AACCTGGTTCCTGAAGATGAGGC	NM_020342	102
	(R)	GATCACGCCTAGCAAGGAAAGC		
S100A4	(F)	CAGAACTAAAGGAGCTGCTGACC	NM_002961.3	126
	(R)	CTTGGAAGTCCACCTCGTTGTC		
S100A16	(F)	GCTCCAGAAAGAGCTGAACCAC	NM_001317007	135
	(R)	ATGCCGCCTATCAAGGTCCAGT		
TMEM25	(F)	GTCCAACCTTCAGCTCAATGACC	NM_032780.4	154
	(R)	CAGCACTGGGAGGCGGATGAA		
TMEM154	(F)	CTAGCCAAGGATCTCAGAGTGC	NM_152680.3	96
	(R)	CCATAACAGAGGGTGTATCTTCC		
TNNT1	(F)	AACGCGAACGTCAGGCTAAGCT	NM_003283.6	140
	(R)	CTTGACCAGGTAGCCGCCAAAA		
TRIM7	(F)	GCCATCTGCGTGGTGTGCGAC	NM_033342.4	144
	(R)	GAACACCTCACAGTCCTCCAGT		
GAPDH	(F)	AAGGACTCATGACCACAGTC	NM_001289745.212	160
	(R)	TTCAGCTCAGGGATGACCTT		
