# Supplementary material for: Gene Expression Profiles Associated with Radio-Responsiveness in Locally Advanced Rectal Cancer
Source: Biology (Basel). 2021 Jun 3;10(6):500. doi: 10.3390/biology10060500 (PMC8226560; doi:10.3390/biology10060500)
Supplement: Supplementary file 1 [file biology-10-00500-s001.zip › Supplemental Table S2. Pyro primer.pdf]

Table S2. Gene pyrosequencing conditions and primers.

Gene Name	Accession Number	Start (genomic)	Strand	Sequence (5'-3')	Tm (°C)	Product (bp)
CTSE	NG_029664.1	4833	Sense	GAGGAGGTGTTAAAGTTTTGAGAGA	61	202
		5012	Anti-Sense	AAACCTACCCAACCCAATCTAAA		
