# Supplementary material for: Gene Expression Profiles Associated with Radio-Responsiveness in Locally Advanced Rectal Cancer
Source: Biology (Basel). 2021 Jun 3;10(6):500. doi: 10.3390/biology10060500 (PMC8226560; doi:10.3390/biology10060500)
Supplement: Supplementary file 1 [file biology-10-00500-s001.zip › Supplemental Table S3. 231 genes.pdf]

Table S3. 231 genes differentially expressed between RR and RS organoids.

	Gene Name	Accession Number	Description
1	AATK	NM_004920	apoptosis-associated tyrosine kinase
2	ACSM3	NM_202000	acyl-CoA synthetase medium-chain family member 3
3	ADAM9	NM_003816	ADAM metallopeptidase domain 9
4	ADGRE2	NM_013447	adhesion G protein-coupled receptor E2
5	AGAP11	NM_133447	ArfGAP with GTPase domain, ankyrin repeat and PH domain 11
6	AGAP2	NM_014770	ArfGAP with GTPase domain, ankyrin repeat and PH domain 2
7	AHNAK2	NM_138420	AHNAK nucleoprotein 2
8	AKAP12	NM_005100	A-kinase anchoring protein 12
9	AKAP7	NM_016377	A-kinase anchoring protein 7
10	AKNA	NM_030767	AT-hook transcription factor
11	ALMS1-IT1	NR_046762	ALMS1 intronic transcript 1
12	ALPP	NM_001632	alkaline phosphatase, placental
13	ALPPL2	NM_031313	alkaline phosphatase, placental like 2
14	ALS2CL	NM_182775	ALS2 C-terminal like
15	AMIGO2	NM_181847	adhesion molecule with Ig-like domain 2
16	ANKRD29	NM_173505	ankyrin repeat domain 29
17	ANKRD44	NM_001195144	ankyrin repeat domain 44
18	ANO1	NM_018043	anoctamin 1
19	ANXA2	NM_004039	annexin A2
20	ANXA3	NM_005139	annexin A3
21	APOE	NM_000041	apolipoprotein E
22	APOLD1	NM_001130415	apolipoprotein L domain containing 1
23	ARHGAP23	NM_001199417	Rho GTPase activating protein 23
24	ARHGAP44	NM_014859	Rho GTPase activating protein 44
25	ARID3B	NM_006465	AT-rich interaction domain 3B
26	ATOH8	NM_032827	atonal bHLH transcription factor 8
27	AXL	NM_021913.5	AXL receptor tyrosine kinase
28	B3GALT5	NM_001278650	beta-1,3-galactosyltransferase 5
29	BACE1	NM_012104	beta-site APP-cleaving enzyme 1
30	BFSP1	NM_001161705	beaded filament structural protein 1
31	BICC1	NM_001080512	BicC family RNA binding protein 1
32	BMP4	NM_130850	bone morphogenetic protein 4
33	C19orf57	NM_024323	chromosome 19 open reading frame 57
34	CACNA1C	NM_001129834	calcium voltage-gated channel subunit alpha1 C
35	CAMK1D	NM_020397	calcium/calmodulin dependent protein kinase ID
36	CAPN6	NM_014289	calpain 6
37	CAV1	NM_001753	caveolin 1
38	CBR3	NM_001236	carbonyl reductase 3
39	CD55	NM_001300904	CD55 molecule (Cromer blood group)
40	CDKN2B	NM_004936	cyclin-dependent kinase inhibitor 2B
41	CDT1	NM_030928	chromatin licensing and DNA replication factor 1
42	CEACAM5	NM_001308398	carcinoembryonic antigen related cell adhesion molecule 5
43	CEACAM5	NM_001308398	carcinoembryonic antigen related cell adhesion molecule 5
44	CECR2	NM_001290046	cat eye syndrome chromosome region, candidate 2
45	CEP78	NM_032171	centrosomal protein 78
46	CGNL1	NM_001252335	cingulin-like 1
47	CHD5	NM_015557	chromodomain helicase DNA binding protein 5
48	COL18A1	NM_130445	collagen type XVIII alpha 1

49	COL6A1	NM_001848	collagen type VI alpha 1
50	CRABP2	NM_001878	cellular retinoic acid binding protein 2
51	CREB3L1	NM_052854	cAMP responsive element binding protein 3-like 1
52	CRIP1	NM_001311	cysteine rich protein 1
53	CTSE	NM_148964	cathepsin E
54	CXCR4	NM_003467	C-X-C motif chemokine receptor 4
55	CXorf57	NM_001184782	chromosome X open reading frame 57
56	DBN1	NM_004395	drebrin 1
57	DES	NM_001927	desmin
58	DKK1	NM_012242	dickkopf WNT signaling pathway inhibitor 1
59	DNAH12	NM_001291661	dynein axonemal heavy chain 12
60	DOC2A	NR_104090	double C2 domain alpha
61	DSC3	NM_024423	desmocollin 3
62	DYNC1I1	NM_001278422	dynein cytoplasmic 1 intermediate chain 1
63	E2F7	NM_203394	E2F transcription factor 7
64	EFR3B	NM_014971	EFR3 homolog B
65	ENPP3	NM_005021	ectonucleotide pyrophosphatase/phosphodiesterase 3
66	ERF	NM_001308402	ETS2 repressor factor
67	EVA1A	NM_032181	eva-1 homolog A, regulator of programmed cell death
68	EVADR	NR_125857	uncharacterized LOC101928353
69	F3	NM_001178096	coagulation factor III, tissue factor
70	FAAH	NM_001441	fatty acid amide hydrolase
71	FAM111B	NM_198947	family with sequence similarity 111 member B
72	FAM117A	NM_030802	family with sequence similarity 117 member A
73	FAM157A	NM_001145248	family with sequence similarity 157 member A
74	FAM83A	NM_032899	family with sequence similarity 83 member A
75	FBXL16	NM_153350	F-box and leucine-rich repeat protein 16
76	FCGBP	NM_003890	Fc fragment of IgG binding protein
77	FER1L6	NM_001039112	fer-1 like family member 6
78	FFAR4	NM_181745	free fatty acid receptor 4
79	FGF19	NM_005117	fibroblast growth factor 19
80	FKBP10	NM_021939	FK506 binding protein 10
81	FKBP1A	NM_001199786	FK506 binding protein 1A
82	FLNC	NM_001458	filamin C
83	FOXD1	NM_004472	forkhead box D1
84	FREM2	NM_207361	FRAS1 related extracellular matrix protein 2
85	FURIN	NM_002569	furin, paired basic amino acid cleaving enzyme
86	FUT1	NM_000148	fucosyltransferase 1 (H blood group)
87	FUT8	NR_038167	fucosyltransferase 8
88	GBP1	NM_002053	guanylate binding protein 1
89	GCNT3	NM_004751	glucosaminyl (N-acetyl) transferase 3, mucin type
90	GJB5	NM_005268	gap junction protein beta 5
91	GPC3	NM_001164618	glypican 3
92	GPR161	NM_153832	G protein-coupled receptor 161
93	GPR63	NM_001143957	G protein-coupled receptor 63
94	GPRIN3	NM_198281	GPRIN family member 3
95	GPSM1	NM_001145638	G-protein signaling modulator 1
96	H19	NR_131223	H19, imprinted maternally expressed transcript (non-protein coding)
97	HAL	NM_001258333	histidine ammonia-lyase
98	HIC2	NM_015094	hypermethylated in cancer 2
99	HPGD	NM_000860	hydroxyprostaglandin dehydrogenase 15-(NAD)

100	IFITM10	NM_001170820	interferon induced transmembrane protein 10
101	IGF2	NM_001291861	insulin like growth factor 2
102	IGFN1	NM_001164586	immunoglobulin-like and fibronectin type III domain containing 1
103	IL18	NM_001562	interleukin 18
104	IL1RN	NM_173843	interleukin 1 receptor antagonist
105	IMPACT	NM_018439	impact RWD domain protein
106	IPO5P1	NR_103742	importin 5 pseudogene 1
107	KIAA1257	NM_020741	KIAA1257
108	KIZ	NM_001163022	kizuna centrosomal protein
109	KLHL29	NM_052920	kelch like family member 29
110	KRT8	NM_001256293	keratin 8
111	LAMA3	NM_198129	laminin subunit alpha 3
112	LGMN	NM_005606	legumain
113	LIF	NM_001257135	leukemia inhibitory factor
114	LIMCH1	NM_001289124	LIM and calponin homology domains 1
115	LINC00674	NR_027418	long intergenic non-protein coding RNA 674
116	LIPH	NM_139248	lipase H
117	LPP	NM_001167672	LIM domain containing preferred translocation partner in lipoma
118	LYPD3	NM_014400	LY6/PLAUR domain containing 3
119	MAGIX	NM_024859	MAGI family member, X-linked
120	MECOM	NM_001105078	MDS1 and EVI1 complex locus
121	MEGF6	NM_001409	multiple EGF like domains 6
122	MIR570	NR_030296	microRNA 570
123	MMD	NM_012329	monocyte to macrophage differentiation associated
124	NANS	NM_018946	N-acetylneuraminate synthase
125	NKD2	NM_001271082	naked cuticle homolog 2
126	NMU	NM_006681	neuromedin U
127	NOS3	NM_000603	nitric oxide synthase 3
128	NOTCH3	NM_000435	notch 3
129	NPC1	NM_000271	NPC intracellular cholesterol transporter 1
130	NRCAM	NM_001193584	neuronal cell adhesion molecule
131	NUPR1	NM_001042483	nuclear protein 1, transcriptional regulator
132	OBSCN	NM_001098623	obscurin, cytoskeletal calmodulin and titin-interacting RhoGEF
133	OGFRL1	NM_024576	opioid growth factor receptor-like 1
134	OLFM2	NM_001304347	olfactomedin 2
135	OXGR1	NM_080818	oxoglutarate receptor 1
136	PARM1	NM_015393	prostate androgen-regulated mucin-like protein 1
137	PART1	NR_024617	prostate androgen-regulated transcript 1 (non-protein coding)
138	PDGFRB	NM_002609	platelet derived growth factor receptor beta
139	PDZRN3	NM_001303139	PDZ domain containing ring finger 3
140	PEAR1	NM_001080471	platelet endothelial aggregation receptor 1
141	PHLDB2	NM_001134437	pleckstrin homology like domain family B member 2
142	PITX2	NM_001204398	paired like homeodomain 2
143	PLAUR	NM_001005376	plasminogen activator, urokinase receptor
144	PLSCR4	NM_001128306	phospholipid scramblase 4
145	PLXND1	NM_015103	plexin D1
146	PMEPA1	NM_020182	prostate transmembrane protein, androgen induced 1
147	PNMA1	NM_006029	paraneoplastic Ma antigen 1
148	PRKAR2B	NM_002736	protein kinase cAMP-dependent type II regulatory subunit beta
149	PRRX1	NM_022716	paired related homeobox 1
150	PRSS22	NM_022119	protease, serine 22

151	PRSS33	NM_152891	protease, serine 33
152	PSCA	NR_033343	prostate stem cell antigen
153	PSIP1	NM_033222	PC4 and SFRS1 interacting protein 1
154	QPCT	NM_012413	glutaminyl-peptide cyclotransferase
155	RAB3B	NM_002867	RAB3B, member RAS oncogene family
156	RASL11A	NM_206827	RAS like family 11 member A
157	REG4	NM_032044	regenerating family member 4
158	RGMB	NM_001012761	repulsive guidance molecule family member b
159	RGS19	NM_001039467	regulator of G-protein signaling 19
160	RHBDF2	NM_024599	rhomboid 5 homolog 2 (Drosophila)
161	RSAD2	NM_080657.5	radical S-adenosyl methionine domain containing 2
162	RUNX3	NM_004350	runt related transcription factor 3
163	S100A16	NM_080388	S100 calcium binding protein A16
164	S100A2	NM_005978	S100 calcium binding protein A2
165	S100A4	NM_019554	S100 calcium binding protein A4
166	SCART1	NR_002934	scavenger receptor protein family member
167	SDR16C5	NM_138969	short chain dehydrogenase/reductase family 16C, member 5
168	SERPINB1	NM_030666	serpin family B member 1
169	SERPINB5	NM_002639	serpin family B member 5
170	SH3TC2	NM_024577	SH3 domain and tetratricopeptide repeats 2
171	SIM2	NM_005069	single-minded family bHLH transcription factor 2
172	SKAP1	NM_001075099	src kinase associated phosphoprotein 1
173	SLC28A3	NR_037638	solute carrier family 28 member 3
174	SLC2A1	NM_006516	solute carrier family 2 member 1
175	SLC2A10	NM_030777	solute carrier family 2 member 10
176	SLC2A1-AS1	NR_033967	SLC2A1 antisense RNA 1
177	SLC39A10	NM_001127257	solute carrier family 39 member 10
178	SLC4A3	NR_048551	solute carrier family 4 member 3
179	SLC7A7	NR_040448	solute carrier family 7 member 7
180	SMOC2	NM_022138	SPARC related modular calcium binding 2
181	SOCS3	NM_003955	suppressor of cytokine signaling 3
182	SPATA18	NM_001297608	spermatogenesis associated 18
183	SPON2	NM_001199021	spondin 2
184	SPTBN2	NM_006946	spectrin beta, non-erythrocytic 2
185	ST3GAL1	NM_003033	ST3 beta-galactoside alpha-2,3-sialyltransferase 1
186	ST5	NM_213618	suppression of tumorigenicity 5
187	STMN3	NM_015894	stathmin 3
188	SULT1C2	NM_001056	sulfotransferase family 1C member 2
189	SULT2B1	NM_177973	sulfotransferase family 2B member 1
190	SYT8	NM_138567	synaptotagmin 8
191	TCF19	NM_007109	transcription factor 19
192	TFCP2L1	NM_014553	transcription factor CP2-like 1
193	TFF3	NM_003226	trefoil factor 3
194	TGFB1	NM_000660	transforming growth factor, beta 1
195	TGM3	NM_003245	transglutaminase 3
196	TMEM154	NM_152680	transmembrane protein 154
197	TMEM25	NM_032780	transmembrane protein 25
198	TMEM38A	NM_024074	transmembrane protein 38A
199	TNC	NM_002160	tenascin C
200	TNFAIP2	NM_006291	TNF alpha induced protein 2
201	TNFRSF6B	NM_003823	tumor necrosis factor receptor superfamily member 6b

202	TNIK	NM_001161566	TRAF2 and NCK interacting kinase
203	TNK2	NM_001308046	tyrosine kinase non receptor 2
204	TNNT1	NM_001291774	troponin T1, slow skeletal type
205	TPPP	NM_007030	tubulin polymerization promoting protein
206	TRAPPC6A	NM_024108	trafficking protein particle complex 6A
207	TRIM68	NM_018073	tripartite motif containing 68
208	TRIM7	NM_203295	tripartite motif containing 7
209	TRPM5	NM_014555	transient receptor potential cation channel subfamily M member 5
210	TTC39C	NM_153211	tetratricopeptide repeat domain 39C
211	TTC9	NM_015351	tetratricopeptide repeat domain 9
212	UNC13D	NM_199242	unc-13 homolog D
213	UPK3B	NM_182683	uroplakin 3B
214	WDR34	NM_052844	WD repeat domain 34
215	WEE1	NM_003390	WEE1 G2 checkpoint kinase
216	WNT10A	NM_025216	Wnt family member 10A
217	ZBTB7C	NM_001039360	zinc finger and BTB domain containing 7C
218	ZIC5	NM_033132	Zic family member 5
219	ZNF169	NM_194320	zinc finger protein 169
220	ZNF185	NM_001178110	zinc finger protein 185 (LIM domain)
221	ZNF254	NM_001278663	zinc finger protein 254
222	ZNF385A	NM_015481	zinc finger protein 385A
223	ZNF425	NM_001001661	zinc finger protein 425
224	ZNF658	NM_033160	zinc finger protein 658
225	ZNF670	NM_001204220	zinc finger protein 670
226	ZNF702P	NR_003578	zinc finger protein 702, pseudogene
227	ZNF736	NM_001294255	zinc finger protein 736
228	ZNF814	NM_001144989	zinc finger protein 814
229	ZNF93	NM_031218	zinc finger protein 93
230	ZSCAN12P1	NR_024063	zinc finger and SCAN domain containing 12 pseudogene 1
231	ZSWIM5	NM_020883	zinc finger SWIM-type containing 5
