# Supplementary material for: Gene Expression Profiles Associated with Radio-Responsiveness in Locally Advanced Rectal Cancer
Source: Biology (Basel). 2021 Jun 3;10(6):500. doi: 10.3390/biology10060500 (PMC8226560; doi:10.3390/biology10060500)
Supplement: Supplementary file 1 [file biology-10-00500-s001.zip › Supplemental Table S4. gene function.pdf]

Table S4. Functional annotations of candidate genes.

Gene Name	Database Identifier	Description
ADAM9	ENSP00000419446	Disintegrin and metalloproteinase domain-containing protein 9; Cleaves and releases a number of molecules with important roles in tumorigenesis and angiogenesis, such as TEK, KDR, EPHB4, CD40, VCAM1 and CDH5. May mediate cell-cell, cell- matrix interactions and regulate the motility of cells via interactions with integrins; ADAM metallopeptidase domain containing
ALS2CL	ENSP00000313670	ALS2 C-terminal-like protein; Acts as a guanine nucleotide exchange factor (GEF) for Rab5 GTPase. Regulates the ALS2-mediated endosome dynamics; VPS9 domain containing
ANO1	ENSP00000347454	Discovered on gastrointestinal stromal tumors protein 1; Calcium-activated chloride channel (CaCC) which plays a role in transepithelial anion transport and smooth muscle contraction. Required for the normal functioning of the interstitial cells of Cajal (ICCs) which generate electrical pacemaker activity in gastrointestinal smooth muscles. Acts as a major contributor to basal and stimulated chloride conductance in airway epithelial cells and plays an important role in tracheal cartilage development; Belongs to the anoctamin family.
ANXA2	ENSP00000346032	Placental anticoagulant protein IV; Calcium-regulated membrane-binding protein whose affinity for calcium is greatly enhanced by anionic phospholipids. It binds two calcium ions with high affinity. May be involved in heat-stress response. Inhibits PCSK9-enhanced LDLR degradation, probably reduces PCSK9 protein levels via a translational mechanism but also competes with LDLR for binding with PCSK9; Belongs to the annexin family.
ANXA3	ENSP00000264908	Inositol 1,2-cyclic phosphate 2-phosphohydrolase; Inhibitor of phospholipase A2, also possesses anti- coagulant properties. Also cleaves the cyclic bond of inositol 1,2-cyclic phosphate to form inositol 1-phosphate; Annexins
APOE	ENSP00000252486	Apolipoprotein E; Mediates the binding, internalization, and catabolism of lipoprotein particles. It can serve as a ligand for the LDL (apo B/E) receptor and for the specific apo-E receptor (chylomicron remnant) of hepatic tissues; Apolipoproteins
AXL	ENSP00000301178	Tyrosine-protein kinase receptor UFO; Receptor tyrosine kinase that transduces signals from the extracellular matrix into the cytoplasm by binding growth factor GAS6 and which is thus regulating many physiological processes including cell survival, cell proliferation, migration and differentiation. Ligand binding at the cell surface induces dimerization and autophosphorylation of AXL. Following activation by ligand, ALX binds and induces tyrosine phosphorylation of PI3- kinase subunits PIK3R1, PIK3R2 and PIK3R3; but also GRB2, PLCG1, LCK and PTPN11. Other downstream substrate candidates for AXL are CBL, NCK2, SOCS1 and TNS2. Recruitment of GRB2 and phosphatidylinositol 3 kinase regulatory subunits by AXL leads to the downstream activation of the AKT kinase. GAS6/AXL signaling plays a role in various processes such as endothelial cell survival during acidification by preventing apoptosis, optimal cytokine signaling during human natural killer cell development, hepatic regeneration, gonadotropin-releasing hormone neuron survival and migration, platelet activation, or regulation of thrombotic responses. Plays also an important role in inhibition of Toll-like receptors (TLRs)-mediated innate immune response; Belongs to the protein kinase superfamily. Tyr protein kinase family. AXL/UFO subfamily.

CD55	ENSP00000356030	CD55 molecule, decay accelerating factor for complement (Cromer blood group); This protein recognizes C4b and C3b fragments that condense with cell-surface hydroxyl or amino groups when nascent C4b and C3b are locally generated during C4 and c3 activation. Interaction of daf with cell-associated C4b and C3b polypeptides interferes with their ability to catalyze the conversion of C2 and factor B to enzymatically active C2a and Bb and thereby prevents the formation of C4b2a and C3bBb, the amplification convertases of the complement cascade. Inhibits complement activation by destabilizing and preventing the formation of C3 and C5 convertases, which prevents complement damage; Blood group antigens
CTSE	ENSP00000350911	Cathepsin E; May have a role in immune function. Probably involved in the processing of antigenic peptides during MHC class II-mediated antigen presentation. May play a role in activation-induced lymphocyte depletion in the thymus, and in neuronal degeneration and glial cell activation in the brain; Cathepsins
FUT8	ENSP00000353910	GDP-L-Fuc:N-acetyl-beta-D-glucosaminide alpha1,6-fucosyltransferase; Catalyzes the addition of fucose in alpha 1-6 linkage to the first GlcNAc residue, next to the peptide chains in N-glycans; Belongs to the glycosyltransferase 23 family.
GPSM1	ENSP00000392828	Activator of G-protein signaling 3; Guanine nucleotide dissociation inhibitor (GDI) which functions as a receptor-independent activator of heterotrimeric G- protein signaling. Keeps G(i/o) alpha subunit in its GDP-bound form thus uncoupling heterotrimeric G-proteins signaling from G protein-coupled receptors. Controls spindle orientation and asymmetric cell fate of cerebral cortical progenitors. May also be involved in macroautophagy in intestinal cells. May play a role in drug addiction; Belongs to the GPSM family.
IL18	ENSP00000280357	Interferon gamma-inducing factor; Augments natural killer cell activity in spleen cells and stimulates interferon gamma production in T-helper type I cells; Belongs to the IL-1 family.
KIZ	ENSP00000479542	Polo-like kinase 1 substrate 1; Centrosomal protein required for establishing a robust mitotic centrosome architecture that can endure the forces that converge on the centrosomes during spindle formation. Required for stabilizing the expanded pericentriolar material around the centriole; Belongs to the kizuna family.
LGMN	ENSP00000376911	Asparaginyl endopeptidase; Has a strict specificity for hydrolysis of asparaginyl bonds. Can also cleave aspartyl bonds slowly, especially under acidic conditions. Required for normal lysosomal protein degradation in renal proximal tubules. Required for normal degradation of internalized EGFR. Plays a role in the regulation of cell proliferation via its role in EGFR degradation (By similarity). May be involved in the processing of proteins for MHC class II antigen presentation in the lysosomal/endosomal system; Belongs to the peptidase C13 family.
LIPH	ENSP00000296252	Membrane-associated phosphatidic acid-selective phospholipase A1-alpha; Hydrolyzes specifically phosphatidic acid (PA) to produce 2-acyl lysophosphatidic acid (LPA; a potent bioactive lipid mediator) and fatty acid. Does not hydrolyze other phospholipids, like phosphatidylserine (PS), phosphatidylcholine (PC) and phosphatidylethanolamine (PE) or triacylglycerol (TG); Belongs to the AB hydrolase superfamily. Lipase family.

NKD2	ENSP00000296849	Naked cuticle homolog 2 (Drosophila); Cell autonomous antagonist of the canonical Wnt signaling pathway. May activate a second Wnt signaling pathway that controls planar cell polarity (By similarity). Required for processing of TGFA and for targeting of TGFA to the basolateral membrane of polarized epithelial cells; EF-hand domain containing
NMU	ENSP00000264218	Neuromedin U; Stimulates muscle contractions of specific regions of the gastrointestinal tract. In humans, NmU stimulates contractions of the ileum and urinary bladder; Belongs to the NmU family.
NPC1	ENSP00000269228	Niemann-Pick disease, type C1; Intracellular cholesterol transporter which acts in concert with NPC2 and plays an important role in the egress of cholesterol from the endosomal/lysosomal compartment. Both NPC1 and NPC2 function as the cellular 'tag team duo' (TTD) to catalyze the mobilization of cholesterol within the multivesicular environment of the late endosome (LE) to effect egress through the limiting bilayer of the LE. NPC2 binds unesterified cholesterol that has been released from LDLs in the lumen of the late endosomes/lysosomes and transfers it to the cholesterol-binding pocket of the N-terminal domain of NPC1. Cholesterol binds to NPC1 with the hydroxyl group buried in the binding pocket and is exported from the limiting membrane of late endosomes/ lysosomes to the ER and plasma membrane by an unknown mechanism. Binds oxysterol with higher affinity than cholesterol. May play a role in vesicular trafficking in glia, a process that may be crucial for maintaining the structural and functional integrity of nerve terminals; Belongs to the patched family.
NRCAM	ENSP00000368314	NgCAM-related cell adhesion molecule; Cell adhesion protein that is required for normal responses to cell-cell contacts in brain and in the peripheral nervous system. Plays a role in neurite outgrowth in response to contactin binding. Plays a role in mediating cell-cell contacts between Schwann cells and axons. Plays a role in the formation and maintenance of the nodes of Ranvier on myelinated axons. Nodes of Ranvier contain clustered sodium channels that are crucial for the saltatory propagation of action potentials along myelinated axons. During development, nodes of Ranvier are formed by the fusion of two heminodes. Required for normal clustering of sodium channels at heminodes; not required for the formation of mature nodes with normal sodium channel clusters. Required, together with GLDN, for maintaining NFASC and sodium channel clusters at mature nodes of Ranvier; Fibronectin type III domain containing
PRKAR2B	ENSP00000265717	cAMP-dependent protein kinase type II-beta regulatory subunit; Regulatory subunit of the cAMP-dependent protein kinases involved in cAMP signaling in cells. Type II regulatory chains mediate membrane association by binding to anchoring proteins, including the MAP2 kinase.
PSCA	ENSP00000301258	Prostate stem cell antigen; May be involved in the regulation of cell proliferation. Has a cell-proliferation inhibition activity in vitro; LY6/PLAUR domain containing
RUNX3	ENSP00000382800	Polyomavirus enhancer-binding protein 2 alpha C subunit; CBF binds to the core site, 5'-PYGPYGGT-3', of a number of enhancers and promoters, including murine leukemia virus, polyomavirus enhancer, T-cell receptor enhancers, lck, IL-3 and GM-CSF promoters. In association with ZFHX3, upregulates CDKN1A promoter activity following TGF-beta stimulation.

S100A16	ENSP00000357693	Aging-associated gene 13 protein; Calcium-binding protein. Binds one calcium ion per monomer. Can promote differentiation of adipocytes (in vitro) (By similarity). Overexpression in preadipocytes increases their proliferation, enhances adipogenesis and reduces insulin-stimulated glucose uptake (By similarity); Belongs to the S-100 family.
S100A4	ENSP00000357705	Placental calcium-binding protein; S100 calcium binding protein A4; EF-hand domain containing
SIM2	ENSP00000290399	Single-minded family bHLH transcription factor 2; Transcription factor that may be a master gene of CNS development in cooperation with Arnt. It may have pleiotropic effects in the tissues expressed during development; Basic helix-loop-helix proteins
SLC39A10	ENSP00000352655	solute carrier family 39 member 10, Zinc is an essential cofactor for hundreds of enzymes. It is involved in protein, nucleic acid, carbohydrate, and lipid metabolism, as well as in the control of gene transcription, growth, development, and differentiation. SLC39A10 belongs to a subfamily of proteins that show structural characteristics of zinc transporters.
TMEM154	ENSP00000302144	Transmembrane protein 154
TMEM25	ENSP00000315635	Transmembrane protein 25; C2-set domain containing
TNNT1	ENSP00000467176	Troponin T type 1 (skeletal, slow); Troponin T is the tropomyosin-binding subunit of troponin, the thin filament regulatory complex which confers calcium-sensitivity to striated muscle actomyosin ATPase activity.
TRIM7	ENSP00000274773	Tripartite motif-containing protein 7; E3 ubiquitin-protein ligase. Mediates 'Lys-63'-linked polyubiquitination and stabilization of the JUN coactivator RNF187 in response to growth factor signaling via the MEK/ERK pathway, thereby regulating JUN transactivation and cellular proliferation; Belongs to the TRIM/RBCC family.
